# Diverse Caregivers in the United States: Implications for Caregiving Across the Lifespan

**DOI:** 10.1093/geroni/igab046.396

**Published:** 2021-12-17

**Authors:** C Grace Whiting, Rita Choula, Yanira Cruz, Lauren Pongan, Feylyn Lewis

**Affiliations:** 1 National Alliance for Caregiving, Washington, District of Columbia, United States; 2 AARP Public Policy Institute, Washington D.C., District of Columbia, United States; 3 National Hispanic Council on Aging, Washington, D.C., District of Columbia, United States; 4 Diverse Elders Coalition, Diverse Elders Coalition, New York, United States; 5 Independent, Washington, District of Columbia, United States

## Abstract

Caregivers with diverse backgrounds make up an important part of the landscape of caregiving in the US. Their unique experiences have been traditionally under-researched in the field of social sciences and underrecognized by society. To further understand the impact of race, ethnicity, class, gender and sexuality onto caregiving, the National Alliance for Caregiving (NAC) and the AARP Public Policy Institute commissioned an in-depth analysis of the dataset collected from the Caregiving in the US 2020 study, which segments populations based on ethnic and sexual identity, geographic location, and income level. Utilizing survey interviews with 1,392 caregivers in the US, this study found differences amongst the African-American, Latinx, and Asian American-Pacific Islander populations in relation to age, time spent caregiving, ADL/IADLs, caregiving strain and intensity, receipt of formal and informal support, and financial impact. In consideration of the distinct challenges presented by diverse caregiving throughout the lifespan, this presentation will also feature results from a 2020-2021 Diverse Elders Coalition and NAC commissioned study on the unmet caregiving needs in diverse communities. 11 virtual listening sessions were held with 400 caregivers of color, including American Indian/Alaska Native caregivers, and LGBTQ caregivers across the nation. Presentation attendees can expect to learn new insights into the experiences of diverse caregivers, while also gaining a fresh understanding of informal and formal support preferences with a multicultural lens. Finally, this presentation will provide recommendations to further prioritize the needs of historically marginalized caregivers in policy and practice.

